# Breasts and the city: an urban ethnography of infant feeding in public spaces within Cardiff, United Kingdom

**DOI:** 10.1186/s13006-021-00384-2

**Published:** 2021-04-29

**Authors:** Aimee Grant

**Affiliations:** Centre for Lactation, Infant Feeding and Translational Research, Swansea University, Swansea, UK

**Keywords:** Breastfeeding, Breastfeeding in public, Determinants of breastfeeding, Gender based violence, Infant feeding, Misogyny, Patriarchy, Qualitative, Sexualisation of the breast, Urban ethnography

## Abstract

**Background:**

Internationally, women report challenges breastfeeding in public spaces. This study aimed to investigate the social-spatial aspects of public spaces in one UK city, Cardiff, in order to suggest possible barriers and facilitators to breastfeeding in public spaces.

**Methods:**

The study observation location guide prioritised places that had been reported as hostile to breastfeeding or breastfeeding friendly in the existing literature. Data were collected between April and September 2018 at various times of day, in several areas of the city, and included transport (*n* = 4), transport hubs (*n* = 3), high streets (*n* = 4), cafes (*n* = 2), a large city centre shopping complex, comprising of three joined shopping malls and a large city centre department store containing a third café. Low inference field notes were written on an encrypted smart phone and expanded soon after. Data were analysed thematically using deductive codes based on the observation schedule. Additional inductive codes relating to places were added.

**Results:**

Overall, public transport and the city centre were inhospitable environments for those who might need to breastfeed, and even more so for those who need to express breastmilk. The core barriers and facilitators across locations were the availability of appropriate seating coupled with either high privacy or politely unimposing strangers (civil inattention). The one variation to this model arose from the department store café, where civil inattention was not performed and there was low privacy, but breastfeeding occurred anyway.

**Conclusions:**

This research highlights the physical and social barriers to breastfeeding within one urban city centre in the UK and its associated transport links. It is clear that there is an urgent need for change in urban city centres and public transport if countries are to meet their aims in relation to increasing breastfeeding rates. Interventions will need to be multifaceted, accounting for social norms relating to infant feeding as well as changes to the physical environment, policy and potentially legal change. Further research should be undertaken in other countries to examine the extent to which hostile environments exist, and if correcting these could facilitate breastfeeding and reduce gender-based violence.

## Background

It is well established that geographies are not gender neutral. The city of Cardiff has long been a place where misogyny flourishes [1], resulting in women altering their behaviour to avoid male violence [2]. This self-regulation can be viewed within the context of unequal power relations created by society [3], including blaming victims [4], tarnishing their reputations [5], and shaming outspoken “feminist killjoys” [6]. Accounts of the lived experiences of women who breastfeed, including those in a recent integrative review [7], and a special issue on Breastfeeding in Public [8], show that breastfeeding in public spaces in the Global North is experienced as a risky business, due to the potential for misogynistic abuse. Breastfeeding thus requires changes in behaviour, to reduce the likelihood of confrontation from strangers [9]. This is particularly relevant to those who may be marked out as breaking traditional “good mother” stereotypes, including mothers who are working-class [10], teenage [11], Black [12, 13], Indigenous [14, 15] and those who breastfeed longer-term [16]. Many of the women in these marginalised groups decide that they should not, or cannot, breastfeed outside of the home.

Those who breastfeed within public spaces, face a range of negative responses, originating from discomfort in strangers [17], family [18] and partners [19]. This results in a range of attempts to regulate the breastfeeding woman’s behaviour from these different groups. The least invasive of these was staring [20]. The stares of those undertaking surveillance generally resulted in women undertaking a lot of additional mental labour trying to be “discreet” [21]. However, arguably through a process of self-regulation, it did result in some women never breastfeeding in public spaces again [15], or identifying types of locations that they would not breastfeed in [22]. Other women were reported to be “virtually house-bound” because they felt that they could never breastfeed in public [23]. An escalation of stranger intervention was found in the UK, where women reported that they had been tutted at [24], asked intrusive questions [25], and had even been asked to leave premises where they were legally entitled to breastfeed [26]. Finally, women have been reported to staff in some instances, including by women [27] who attempt to uphold the sexualisation of breastfeeding discourse propagated by the patriarchy [28]. However, it should be noted that in a study by Kate Boyer of 57 mostly middle-class women, half of respondents did not report any negative experiences [29]. Accordingly, the literature is missing the experiences of these women who breastfed and did not encounter negative responses.

One area designed to *solve the problem* of breastfeeding away from home is the mother & baby room. The acceptability and utility of these spaces is contested, with some study participants finding them positive, at least in the early weeks [28]. Other users, however, found them confining, dirty, and generally unsuitable for infant feeding [23]. Within a recent qualitative systematic review of breastfeeding in public (Grant et al., in preparation) the only location that was noted as *positive* to breastfeed in was coffee shops in middle-class areas. In the study, reported by Boyer [23, 29], the women who described the pleasant cafes in which to breastfeed did not routinely have access to these coffee shops, reducing their utility significantly. All other public places were identified negatively, with public transport [30] and places where goods were for sale [26], or eating occurred ranking as the most highly offensive to observers [31].

In order to study these phenomena empirically, an ethnographic approach was taken to data collection, situated in a feminist interpretivist paradigm [32]. Principles of “urban ethnography” [33] were adopted within one UK city.

## Methods

### Aim

To empirically study the urban environment in Cardiff, UK in relation to barriers and facilitators for breastfeeding in public, in order to inform future interventions.

### Setting

The city of Cardiff in south Wales, UK was chosen as the area for observations. One of the primary factors was the strong relationship between public health researchers, the Welsh Government who have devolved power to manage all aspects relating to health, and the Welsh national public health agency, Public Health Wales [34]. This has led to many successful feasibility studies, including in relation to smoking [35], infant feeding [36] and reducing alcohol related harm in the city centre environment [37].

Data collection sites were prioritised based on the positive and negative places identified in the literature. Locations studied were: public transport, the city centre (malls and high streets), suburban coffee shops, and mother and baby rooms. Settings were required to be open to members of the public, excluding spaces such as nurseries and mother and baby groups. All data were collected between April and September 2018.

### Design and data generation procedure

Walking and movement were central to the embodied data collection experience [38]. This involved wondering with a purpose and recording what was seen, paying attention to sensory factors and the physical environment primarily, as well as social interaction. Breastfeeding in public was treated as a deviant behaviour in the UK at the time of data collection, and the researcher considered how it would feel to be breastfeeding a hungry baby in a range of spaces with varying degrees of privacy.

Observations were undertaken by the author. The author carried a notebook, pens, smart telephone and digital recorder in her “ethnographic handbag” with each tool accessible at short notice. The observation guide (see Table 1) was stuck in the back of her notebook, to allow for easy access when required. Prior to beginning data collection, the author undertook training from leading authorities in ethnography [39] Sara Delamont [40] and Paul Atkinson [41] in constructing fieldnotes prior to beginning her data collection. Field notes were collected in a low-inference style, recording both the physical environment and social encounters observed. The aim of this field note style was to allow for the majority of the interpretation to occur at the point of expansion and analysis. During data collection, it became apparent that I was noticeable using a notebook to record my thoughts. At this point I transferred data collection to the Microsoft Word application on my encrypted smart telephone, which was linked to the University’s secure network. In doing so, I became invisible, a part of the scenery, except when taking photos, or sketching maps of the spaces I was in.

**Table 1:** Observation guide

Theme	Sub-theme
**People**	Mothers
	Observers (general)
	Observers (members of staff)
	Babies
	Those with mothers
**Places**	Designated places to BF outside of the home
	Lack of space to breastfeed outside of the home
	Breastfeeding in non-designated places
	Spaces you don’t breastfeed in
**Social norms**	Understanding the legal position
	Sexualisation of breasts
	Breastfeeding in public as anti-social
	Formula feeding culture
	Breastfeeding in public as NOT offensive

Prior to beginning an observation and following observations, the researcher “checked in” using a lone worker safe call procedure, as recommended for lone researchers [42]. The study was granted ethical approval by Cardiff University School of Medicine ethics committee. Alongside this, a formal qualitative data collection and analysis plan was written and agreed by the internal Centre for Trials Research Quality Assurance team.

### Researcher reflexivity

The researcher is a white gender-apathetic (gender-meh) woman who was in her mid-30s at the time of data collection. She is from a working-class background and lived for 5 years in the working-class area used within the research. However, she was 8 years post-doctorate at the time of data collection, married to a middle-class man, living in secure housing and was earning above the median income level for the UK. Accordingly, she was able to pass as both working-class and middle-class during data collection, and used clothing as a way of changing her external identity [40].

The researcher did not have any children at the time of the research, however she had considerable experience, accumulated over 5 years, of researching motherhood using mostly qualitative methods. The researcher has expertise in ethnographic data collection (see for example: [43]). Furthermore, she believes her observational skills are enhanced by being Autistic, as Autistic individuals who may commonly be believed to be “high functioning” (a label that is no longer viewed as appropriate) spend considerable time and effort studying others, in an effort to pass as neurotypical in ableist societies in the Global North [44].

### Data analysis

As soon as possible after data collection, and generally on the same day, the field notes were expanded on, to ensure important details were retained. In doing so, early analytical thoughts were added to documents. A period of immersive familiarisation occurred over the full data collection period, where field notes were returned to in order to contextualise later experiences. Furthermore, notes were made frequently outside of data generation periods, containing thoughts on what had been seen and their links to the literature or a relevant experience outside of a formal data collection period. Data were analysed in May–August 2020, using reflexive thematic analysis [45]. Analysis was conducted “by hand”, an approach that the researcher felt brought her closer to the data, without using a Computer-Assisted Qualitative Data analysis software to perform data management skills for her [46]. The topics from the observation schedule were used as deductive codes, and additional inductive codes were added where required.

## Results

The results section begins by providing an overview of the data collected. The data is then presented in relation to themes arising from individual locations, to allow for the greatest potential impact from those designing public spaces. Theoretical concepts will be returned to within the discussion.

### Summary of data

Data were collected from 14 locations in and around Cardiff, as well as a second train station in Bristol (total *n* = 15). The majority of these locations could be further sub-divided, as is shown in Table 2.

**Table 2:** Data collection locations

Category	Location	Sub-locations
**Transport: vehicles** **(*n*** **= 4)**	Taxi	–
	Train (mainline, Swansea – London)	Carriage; Toilets
	Bus (City centre to University Hospital of Wales)	Bus Stops; Bus (Cardiff Bus Company)
	Aeroplane (Flybe, economy)	Cabin; Toilets
**Transport: hubs** **(*n*** **= 3)**	Cardiff Railway Station	Back entrance; Parent & Baby Room (not found); Toilets; Café; Platforms (including benches); Waiting room; Front entrance
	Bristol Parkway Railway Station	Platform; Waiting room
	Cardiff International Airport	Check in area; Special Assistance area (within check in area); Security; Departure area including: Departure lounge, Costa coffee, Ritazza café/bar, Toilets, Baby changing room, Duty free shops, Departure gate
**High Streets: outside of city centre (*n*** **= 3)**	Cathays (student area)	Outside library, Supermarket (no toilets), Outside shops
	Whitchurch (middle-class area)	Outside shops
	Splott (working-class area)	Outside shops, Supermarket (no toilets)
**High street: main city centre** **(*n*** **= 1)**	Queens Street, The Hayes	Benches, Outside shops
**Cafés** **(*n*** **= 2)**	Whitchurch (middle-class area)	Outside seating, Downstairs, Upstairs, Toilets
	Splott (working-class area)	Inside seating, Toilets
**Shopping mall: city centre** **(*n*** **= 1)**	Queens Arcade; St David’s Centre, St David’s 2 (interconnected)	Benches (multiple locations), Outside shops (including two underwear shops), Toilets, Baby changing, Infant feeding room, Family toilets (with 2 toilets per cubicle), elevators (with sexualised underwear advertising)
**Department store** **(*n*** **= 1)**	John Lewis	Shop floor, Toilets, Mother and Baby area, Café (noted as a safe place to BF on Feed Finder app)

### Thematic analysis

Within the analysis, six key areas are described: public transport, urban shopping malls (large indoor shopping centres containing multiple businesses), high streets (the main street in a town or area, containing shops, banks and other businesses), mother & baby rooms, toilets and cafes. Within each of these, the common overarching theme of an available and appropriate seat will be provided.

#### Public transport: seats on planes, trains and automobiles

For passengers with tickets, places to sit were available at transport hubs or on public transport. Railway station waiting rooms were not particularly good quality in terms of comfort or practicality for those travelling with infants. Furthermore, they seemed to be governed by social norms requiring a certain kind of behaviour, as I summed up in my notes:

*The waiting room is rather small, drafty … and physically not ideal for breastfeeding. Four rows of cold metal seats are provided, each facing one other row. The arm rests on each individual chair are not well positioned for breastfeeding, and they may be shared with an adjacent stranger … Most people in the room are doing “civil inattention”. Lots are using smart phones and staring at the screen, one older couple are chatting quietly, others reading books/a newspaper. Although the announcements are loud in the room, the people are quiet, like a library. It feels as if there is an unspoken rule to be “quiet and considerate.” (Fieldnotes: Cardiff Railway Station Waiting Room: platform 1).*

At Cardiff International Airport, the small number of seats in the check in area, were similarly robust, cold, metal and including arm rests. It was not possible to use a seat provided and check in simultaneously, because of this several passengers in the check in queues were sat on their luggage or the floor, which was uncarpeted and cold. One contrasting handful of more comfortable, vinyl covered padded seating in the arrival hall was marked for passengers requiring “special assistance”. However, these needed to be booked with the airline in advance, and their use was policed by a member of staff sat in a cubicle beside the seats. This type of vinyl covered padded metal seats was available in large numbers in the *departure lounge*, allowing plenty of vacant seats between passengers. Alongside these, “two ‘statement’ benches (with no backs) shaped like a child’s interpretation of a snake (a wiggly curved line)” were situated outside of the toilets, aiming to provide seating that was used in a more short-term way.

The quality of seats on the transport itself was variable. I found the bus seats to be itchy through denim jeans, and the seats on the plane and train to be more comfortable, but narrow:

*With my arms against my sides I am pressed into the wall of the train but I take over about 5-10cm of the neighbouring chair. It would be very difficult to breastfeed with a stranger next to you … (Fieldnotes: train Cardiff – Bristol)*

Within the taxi, the seating was comfortable and spacious. However, I was aware of the legislation regarding babies not being in arms, and I also felt the gaze of the male taxi driver in the rear view mirror as we were having a conversation on the journey.

#### Shopping malls and department stores: highly visible seating

In both the complex of multiple joined shopping malls and the department store, some indoor seating was provided without charge to those who felt comfortable or able to enter those semi-private spaces. Within the complex of three shopping malls, there were a range of different types of seat. First, in the older part of the mall (St David’s Centre), wooden benches, some divided into individual seats by metal arms, were situated in the middle of the walkways between shops. These were placed in rows of two, which were back-to-back. I noted: “very busy (pedestrian traffic); people walking past have to get very close to those seated … I sensed that for those who feel vulnerable … the combination of height difference and proximity could be intimidating”.

In the new area of the shopping mall (St David’s 2), I noted that alongside flat wooden benches with very low backs and metal arm rests downstairs, “some comfier, padded vinyl-covered benches” were provided in the upstairs atrium, although these did not have backs available to rest against. Attempts had been made to put these away from the main footfall, and far fewer people walked past me as I sat on these benches. However, the more open space in the upstairs of the shopping mall, to allow for multiple sets of escalators, aided by all “walls” being made of clear glass, meant that I felt more “exposed” to public scrutiny whilst sitting there.

#### High streets: uncomfortable seats

This was the only type of completely free to access seating open to everybody in the study; none of it felt like a facilitator to infant feeding. First, considering the three high streets outside of the city centre, there was a lack of seating available. Within the working-class high street, there was no seating available. This was a surprise, as it had contained a large quantity of benches (more than 10) when I lived there from 2012 to 2016, which were used heavily. Likewise, in the student area high street, there was no seating, although slightly beyond the high street, there was a small garden outside a library with benches and concrete individual seats. The middle-class high street had wide pavements and plenty of space, compared to the student area but only three rotten wooden benches with no arm rests.

Within the city centre, the wooden benches on *Queen Street* were divided into individual seats using metal arm rests; these have been identified as poor quality in relation to comfort by *Rate This Bench* [47]. Alongside these utilitarian anti-homeless benches, in *The Hayes* area of the city centre, there was a range of outdoor seating available; cold black stone cuboids, aiming to seat two people and long curved black stone backless benches, similar in shape to the curved benches within the *departure lounge* of Cardiff International Airport.

By far the most regular form of seating around the city which was accessible to all was in the form of bus stops. However, these seemed designed to *prevent* comfort and stability, and would not easily allow for breastfeeding:

*The bus stops have small rounded (convex) red plastic “benches” to rest against, but they’re not really aimed to be sat on, just uncomfortably perched! (Field notes: high street in student area)*

#### Mother and baby rooms: not enough seats

Mother and baby rooms were few and far between, and were either inaccessible or not present at the two Cardiff based transport hubs. At Cardiff Central Railway Station, I saw a single sign to a “parent & baby room”. I tried to find the room, but only found nappy changing facilities within a toilet cubicle. It was not possible to find information relating to space for infant feeding on either the Great Western Railway or Transport for Wales websites. When I contacted Transport for Wales by email, they told me that there was a mother and baby room that was kept locked, requiring a guard to access the room. Upon further questioning, I was told that the room contained a chair and plug. The lack of signage and visibility of the room means that it is not particularly useful to those who are ‘not in the know’.

In relation to Cardiff International Airport, I found information on the internet stating that there was a mother and baby room “on 1^st^ floor departures” at the Airport, but that women are “welcome to feed wherever”. However, I was unable to find anything other than a (smelly) baby changing room, which did not contain a chair or plug to facilitate expressing. I asked a member of staff for help finding space to breastfeed:

*Staff: What is it for, just privacy? No sorry I don’t think so, we’re really behind the times here.*

*She goes off to ask someone and asks me to wait where I am … .About 15 minutes later I see her organising stock in the shop and she looks over and smiles at me. She doesn’t come to let me know either way about the mother and baby room. I guess it doesn’t exist.*

Both the shopping mall and department store had mother and baby rooms. Within the shopping mall, the mother and baby room was located near the toilets. It was identifiable by an image of a baby’s bottle on the door, and felt very unwelcoming. The room was cold, smelt of baby faeces (due to the changing station located in the room), and the only seating was a single small, angular, two-seater black leather sofa with the arms at the same height as the back. The room did not allow for more than one person to comfortably sit at a time. This was in contrast to the six “family bathrooms”, containing an adult and child toilet, but no seat for infant feeding.

The final mother and baby room was the one frequently described by mothers in other research projects I have worked on as “the best” in the city centre; hosted within the third floor of the John Lewis Department store. The mother and baby room was also located near the toilets, but separate to them, and according to images and descriptions found online, contained a separate area defined as a “Breast feeding area”, alongside a “bottle feeding area” with bottle warming facilities. When I arrived at the mother and baby area, I could hear crying inside. Not knowing how the room was laid out, I did not want to disturb somebody who was struggling to feed, so I sat outside the room, on one of the three chairs provided in the corridor. I waited for 20 min, but nobody came out of the room and two more mothers with babies went in. It appears that the room was so much better than other facilities, that it was heavily over-used, even on a weekday at lunchtime.

#### Toilets: dirty seats

Toilets were visited in the transport hubs, on public transport, and in cafes. The worst toilets were on public transport: they were cold and industrial in design, urine scented, dirty and cramped. These were closely followed by the toilets at Cardiff Central Railway Station, which “were really dirty; the floor is visibly dirty … with an overflowing sanitary bin and various pieces of rubbish left …” The toilets at the airport, by contrast, were unremarkable; reasonably clean and tidy.

This theme of OK, but not outstanding toilets continued. The toilets in the shopping mall: “smell clean, but doesn’t look spotless. The floor … looks a bit dirty …” . The John Lewis toilets and department store toilets were similar. The toilets within the middle-class café were not particularly clean, heavily scented with air freshener and were oppressively dark. By contrast, the toilet in the working-class café was inexpensively decorated but “spotlessly clean”.

#### Cafes: paying for a seat

The Feed Finder app [48], developed by the University of Newcastle Upon Tyne to support mothers to identify “Breastfeeding Friendly Places”, returned only one result for Cardiff: the John Lewis café. At first glance, it did not feel ideally suited for breastfeeding in the UK, as it was a “big and open” space with bright lights and low privacy. That said, there was a high degree of civil inattention, and it was not possible to be seen by external observers, due to the café being situated on the third floor, and having the windows covered with a design to prevent external viewers. This coupled with a reputation for being “a safe space” may have resulted in a large number of babies being present. During my visit a woman next to me, with a table’s gap between us, on a long bench containing five smaller tables, breastfed her infant. In the hour I was in the café, two other women breastfed their babies, and I saw a woman ask a member of staff to warm milk in a baby’s bottle, which she did quickly and without complaint, despite my feeling that: “It doesn’t look as though they have time to be attentive (but they are).”

The middle-class café was akin to the positive place in Boyer’s (2011) study [23]. The layout encouraged a feeling of intimacy: different furniture and lighting was used at most tables, including a range of sofas, arm chairs and wooden high chairs. The customers were a mixture of families with young children and/or babies, and couples in their 30s. The staff were polite and customers behaved in individualistic ways, including a customer doing embroidery and parents feeding children items not purchased on the premises. One woman was breastfeeding a baby in an area that was both hidden from most customers’ view, but highly visible to those entering and leaving.

The café in the working-class area had a totally different feel to it. The décor was clean, bright and airy, with a “counter” where staff members could see all tables, encouraging a feeling of potential surveillance. The tables were all identical in design, with narrow modern chrome and faux leather dining chairs. This less expensive café, in contrast to the other two cafes, did not encourage lingering with an expensive drink, but relied upon a faster turnover of customers. During the observation, on a weekday, the space felt quite macho, with many tables occupied by tradesmen in work wear and high visibility jackets having lunch and reading tabloid newspapers, some of which still displayed sexualised naked breasts on a daily basis. No babies or children were present, which in my previous experience was common.

At all three transport hubs, cafes were available. The pace of turnover was high, resulting in a feeling of busyness and slight stress from customers and staff alike. Gaps between tables were small, which would make fitting a pushchair into the space challenging, and would result in a high level of visibility for those who were breastfeeding.

## Discussion

This research examined a range of locations in one UK city in order to determine physical and social barriers and facilitators to breastfeeding. In general, the city centre was an inhospitable environment for those who might need to breastfeed, and even more so for those who needed to express breastmilk [15]. Mother and baby rooms are suggested by critics of public breastfeeding to be the appropriate place to feed infants, however, the two mother and baby rooms in this study were either poor quality [23], or had a lack of available space [28]. Unsurprisingly, none of the toilets investigated was in the least bit suitable for breastfeeding, particularly in relation to cleanliness [49]. The core barriers and facilitators running through the data appeared to be the availability of appropriate seating coupled with either high privacy or civil inattention [50] from other users of the space. The one variation to this model arose from the department store café, where civil inattention was not necessarily performed, and there was low privacy. Accordingly, this outlier suggests that there is the potential for culture change within the UK which would reduce the stigma of breastfeeding in public places.

Within the findings, a number of theoretical concepts are relevant. In the vast majority of locations, plenty of opportunities were available for patriarchal surveillance by strangers, as identified by Foucault (1991) [3] and accompanying “self regulation” among would be breastfeeders [5]. A second opposing set of theoretical concepts were included in relation to observers who chose not to perform surveillance by enacting polite “civil inattention”, which was sometimes interrupted by babies being “interactionally open” when looking at those around them, resulting in an interaction between strangers [51]. Whilst this civil inattention might appear positive on the face of it, it could be oppressive, as social norms dictate what “polite” behaviour is, including ensuring children are quiet in public.

Needing to breastfeed on public transport is a frequently stated concern of those who are considering how to feed their baby outside of the home [30]. During the fieldwork, the high potential for surveillance on buses, accompanied by a lack of space on trains and aeroplanes confirmed that it would be challenging to breastfeed in these places due, particularly if you were not confident breastfeeding around strangers [30]. It is not surprising, therefore, that breastfeeding was not observed on public transport, and that babies themselves were relatively uncommon. Breastfeeding in exposed outdoor areas has also been reported to be unpleasant to women who are marginalised due to being working-class [25]. The seating options available on the four high streets in the study were low in number, poor quality, and had high potential for surveillance. None of these factors make them an inviting place for women to breastfeed.

It can be seen that breasts, and by extension breastfeeding, remain a sexualised spectacle in the UK [52], with highly sexualised advertising, identified as problematic decades ago (see for example: [53]), present in the shopping mall. Furthermore, large pieces of advertising were particularly visibile to those using elevators, as is common when travelling with infants, as all of the external lift doors were covered in underwear advertisements. Alongside this, one of the two mother and baby rooms, positioned in close proximity to one set of elevators, was indicated by an image of a bottle alone, reflecting entrenched cultures relating to formula feeding and the inappropriateness of maternal breasts existing in close proximity to sexual (and profit generating) breasts [54].

The two places where people appeared to be able to breastfeed without fear of being perceived as exposing a sexual breast were both middle-class cafes. The cafe within the middle-class area, a location highlighted by Boyer [29] as breastfeeding friendly, afforded intimately divided ‘zones’, accompanied by civil inattention from other customers and attentive but discreet staff. By contrast, the John Lewis café had very high visibility to other café users, tables close to each other (including the use of shared benches) and not all customers performed polite civil inattention [51]. These interactions directed towards breastfeeding mothers can be experienced as positive, as reported by half of Boyer’s (2012) middle-class participants, but they may feel intrusive, judgemental and rude, particularly to marginalised groups [21]. The third café, in the working-class area allowed for constant surveillance from staff, customers inside and pedestrians outside [3], and the sexual breast was present through the reading of tabloid newspapers, which was not seen in other locations.

Theorising the acceptability, or stigmatisation, of breastfeeding in a location with high potential for surveillance, social class may be relevant. It may be that a largely middle-class customer base results in the attitude that everyone is assumed to know how to behave in “good taste”. This is in contrast to class-based surveillance arising from disgust directed to marginalised, including working-class communities [55]. Marginalised groups themselves practice stigmatising behaviours more commonly than their non-marginalised counterparts, in order to distance themselves from the undesirable, demonised “other” identified by the media and politicians [56].

This research was undertaken in one UK city over several months, using a range of areas including the city centre and various high streets. Low inference field notes accompanied by analytical thoughts added at a later stage aimed to record the challenges within the environment under study in a clear. However, it cannot be said to be representative of the UK as a whole. Future research could concentrate on places where breastfeeding in public is the norm, using salutogenic, asset-based, case studies to understand the environmental, social and political factors that have contributed to the creation of pro-breastfeeding space. Furthermore, whilst the author has conducted a large body of work on experiences of pregnancy and infant feeding, the author was not a breastfeeding mother herself, and this may have impacted on subtle nuances that were or were not identified.

## Conclusions

More appropriate spaces and a more breastfeeding-supportive society are urgently needed to prevent exposure to misogynistic attitudes towards breastfeeding. This would enable those breastfeeding to expend less energy on performing “socially sensitive lactation” [57], making leaving the house with an infant less burdensome. In turn, mothers would experience less anxiety about breastfeeding in public, and accordingly less self-regulation. Overall, this process would normalise breastfeeding in a wider range of public spaces, and hopefully result in an increase in breastfeeding rates. However, for this to happen, there is an urgent need to make existing public spaces more breastfeeding friendly. In addition to environmental change, normalising breastfeeding will reduce the discomfort that observers feel when they see breastfeeding.

## Data Availability

The data that support the findings of this study are available from the author but restrictions apply to the availability of these data, which were used under license for the current study, and so are not publicly available. Data are however available from the authors upon reasonable request and with permission of the Centre for Trials Research, Cardiff University, a registered Clinical Trials Unit.
